# Primary Anti-Phospholipid Antibody Syndrome: Real-World Defining Features of Rethrombosis in the Course of Disease

**DOI:** 10.1155/2022/7331586

**Published:** 2022-11-10

**Authors:** Maria Francisca Moraes-Fontes, Filipa Pedro, Maria Manuel Campos, Melissa Fernandes, Sule Yavuz, Francisco Oliveira, António Panarra

**Affiliations:** ^1^Unidade de Doenças Auto-Imunes/Medicina 7.2, Hospital de Curry Cabral, Centro Hospitalar Universitário de Lisboa Central, Lisbon, Portugal; ^2^Serviço de Medicina, Hospital Distrital de Santarém, Santarém, Portugal; ^3^Laboratório de Hemostase, Serviço de Imuno-Hemoterapia, Hospital de Curry Cabral, Centro Hospitalar Universitário de Lisboa Central, Lisbon, Portugal; ^4^Department of Medical Sciences, Rheumatology, Uppsala University, Uppsala, Sweden; ^5^Champalimaud Research, Champalimaud Foundation, Lisbon, Portugal

## Abstract

**Objective:**

We aimed to identify features that allow differentiation of primary antiphospholipid syndrome (PAPS) patients that suffer recurrent thrombotic events (RTE) despite anticoagulation, from the other diagnosed PAPS patients.

**Methods:**

This was an exploratory study of anticoagulated PAPS patients attending an Autoimmune Diseases Unit (1998-2018). From 2016, anti-phospholipid antibodies and lupus anticoagulant were determined for each patient at consecutive visits, collected together with retrospective clinical characteristics, laboratory, and therapeutic markers and compared according to the occurrence of thrombotic events during follow-up.

**Results:**

Overall, two thirds of the patients were female, 93% were Caucasian, with a median age of 40 years at diagnosis, for a median time of 11.5 years in follow-up. Out of 54 patients, 10 were identified with RTE. There were no significant differences among the RTE and non-RTE patients as far as classical risk factors for clotting disorders. The RTE group was characterized by a higher proportion of younger patients, male sex and positivity for all laboratory markers, and initially and over follow-up as well as a sustained high-risk profile based on APS laboratory markers. Anticardiolipin IgG at onset was the only statistically significant marker of the RTE group. At the end of follow-up, consistent reversion to negative status was a rare event, observed in 20% of RTE vs. 25% of non-RTE patients.

**Conclusions:**

Despite therapy, we were able to identify features associated to thrombotic events in patients with PAPS. Prospectively regular clinical and laboratory monitoring might be warranted in order to treat APS more assertively.

## 1. Introduction

The diagnosis of thrombotic primary antiphospholipid syndrome (PAPS) has severe implications as lifelong anticoagulation with vitamin K antagonists (VKA) is recommended [1]. Anticoagulation is almost never stopped due to fear of thrombotic recurrence [2], safe interruption having been rarely described in seronegative patients [3].

Even though an optimal TTR is reported to be associated with the most successful patient outcomes [4], it should be emphasized that VKA have a very narrow therapeutic range. Frequent monitoring through the international normalized ratio (INR) to determine the optimal dose is required. Indefinite anticoagulation is a lifetime burden and, not surprisingly, PAPS patients have a poor quality of life [5]. Advances in warfarin pharmacogenomics which determine the initial response to therapy are not applied in routine clinical practice [6]. Even if a stable INR is rapidly achieved, frequent blood draws for INR control due to warfarin interactions with drugs and diet are still required. Self-testing using portable INR monitoring devices has not emerged as a convenient alternative [7]. For all of these reasons, it is not unreasonable to believe that INR control is frequently suboptimal in most anticoagulated patients.

PAPS diagnosis is supported by exclusion of hereditary thrombophilia and a moderate to high-risk profile, based on several combinations of anti-phospholipid antibody (aPL) tests and titers with testing on two occasions, at least 12 weeks apart. A high-risk profile, associated to a greater risk for thrombotic events, has been defined as “The presence of lupus anticoagulant, or of double (any combination of lupus anticoagulant, anticardiolipin antibodies or antibeta2 glycoprotein I antibodies) or triple (all three subtypes) aPL positivity, or the presence of persistently high aPL titres” [8].

Recently, a larger panel of auto-antibodies and clinical features have been highlighted as contributors for a PAPS diagnosis [9], but these do not benefit previously diagnosed patients. The global APS score (GAPSS) as well as aGAPSS have helped stratify patients with a high probability of developing recurrent thrombosis [10]. There is however, no information as regards the utility of their consecutive application for risk profile adjustment or to inform on disease activity for individual patients. Apart from the aPL profile, smoking, hypertension, and thrombocytopenia are independent risk factors for the development of thrombosis in aPL carriers [11]. Nonetheless, to date, there is not enough data to issue recommendations on primary prophylaxis, independently of score values or risk profiles in otherwise healthy patients [12, 13].

Currently, after an APS diagnosis, no further laboratory testing is required. However, using Sapporo criteria [14], PAPS overdiagnosis due to overinterpretation of laboratory tests is common. Lack of standardization of phospholipids-dependent coagulation assays (aPL tests) and interference with the lupus anticoagulant (LA) assay, [15] conformational changes in beta (2)-glycoprotein 1 (*β*2-GP1) potentially resulting in transient auto-antibodies [16], and commercial tests unable to identify the subpopulation of pathogenic antibodies [17] are, altogether, potential causes of an erroneous PAPS diagnosis.

We aimed to identify features that allow differentiation of PAPS patients who suffered recurrent thrombotic events (RTE) that occurred after establishment of APS diagnosis despite anticoagulation, from the other diagnosed APS patients.

## 2. Methodology

### 2.1. Patients and Therapy

An exploratory study of 67 PAPS patients started in 2016 with a retrospective component between January and June 2019 (pre-COVID pandemic). All patients fulfilled the Sapporo criteria for PAPS diagnosis and were diagnosed between 1997 and 2017, with a follow-up time from diagnosis of at least one year. Demographic and clinical data were documented from patient files including type and number of events at the time of diagnosis and presence of risk factors for thrombotic disease. Control of comorbidities (particularly those that are considered risk factors for thrombosis) and full medication histories throughout follow-up were not recorded. Arterial events such as stroke, myocardial infarct, and mesenteric artery thrombosis as well as pulmonary embolus and portal vein thrombosis were considered potentially life-threatening. RTE were characterized according to number and type (arterial or venous) during follow-up. Therapy consisted of warfarin and/or acenocoumarol for every patient. No haemorrhagic episodes were recorded. None were treated with novel oral anticoagulants. Out of 67, 13 patients were excluded from the present study on the grounds of secondary APS, hereditary thrombophilia, or the pure obstetric form of the syndrome. The study protocol was approved by the Centro Hospitalar Universitário Lisboa Central Ethics Committee (process 183/2015).

### 2.2. Laboratory Tests

In order to monitor therapy, at the time of diagnosis, every patient was enrolled in a designated service, “An Anticoagulation Clinic” set up in the Blood Transfusion Service (Serviço de Imuno-Hemoterapia). Prothrombin-time tests were expressed as INR and performed as a personalized service, in order to maintain the INR between 2.0 and 3.0 (the general recommendation in most patients). An evaluation of percentage of the time in therapeutic range (TTR) was performed for every patient followed in the clinic in the last year of follow-up and considered as follows: good control (>75%), moderate control (60%-75%), or poor control (<60%).

We introduced regular laboratory monitoring from January 2016 to December 2018. Antibody titers (aPL) and LA were measured at consecutive visits, not according to a preestablished schedule. Previous values including those at the time of diagnosis were recorded. Quality control overtime allowed for reproducibility of the test when the commercial assays changed in 2005. From then on, until December 2018, ELISA assays from Euroimmun^®^ were used for the quantification of IgM and IgG antibodies against anticardiolipin (ACA) and anti-*β*2-GP1 antibodies. The upper limit of normal was 12 for ACA IgM (MPL), ACA IgG (GPL), and anti-*β*2-GP1 IgM (MPL) and 20 for anti-*β*2-GP1 IgG (GPL). A minority of patients (*n* = 16) were diagnosed with APS before 2005. The diluted Russell viper venom time-dRVVT (dRVVT Screen/dRVVT Confirm) from HemosIL/Werfen was used with low and high concentration of phospholipids to test for the presence of LA. The result was obtained as a normalized ratio (obtained between the ratios of screen and confirm tests, which in turn were calculated from patient time and the normal control time ratios). When the value was greater than 1.2, the result was positive (weak, moderate, or strong according to the normalized ratio obtained). Of note, when the INR was >1.5 but ≤3.0 the dRVVT involved an extra step consisting of LA negative control mix to validate positive results. If the INR was ≤1.5, the dRVVT result was considered reliable and otherwise not performed if the INR was >3.0. Reversion of positive to negative status at the end of follow-up was defined from the time when repeated aPL and LA were absent, measured on at least two consecutive occasions, at least 12 weeks apart, with a follow-up of at least 1 year.

None of the patients included in this study suffered from hereditary thrombophilic disorders such as Factor V Leiden, Prothrombin G20210A mutation, methylenetetrahydrofolate reductase C677T variant in homozygosity, antithrombin III, and protein S or protein C deficiency, and each was negative for Human Immunodeficiency Virus and hepatitis B and C. After a pulmonary embolus, every patient was carefully followed for the presence of pulmonary artery hypertension. One patient with chronic thromboembolic pulmonary hypertension required endarterectomy [18].

### 2.3. Classification

Every patient was retrospectively classified as low, medium, or high-risk profile at the time of diagnosis and last visit according to laboratory criteria [8, 15]. aGAPSS was also determined at initial visit by adding the points corresponding to hyperlipidaemia (3), arterial hypertension (1), ACA (5), anti-*β*2-GP1 antibodies (4), and the lupus anticoagulant test (4).

### 2.4. Statistical Analysis

Continuous variables were recorded as medians (interquartile range (IQR)), and dichotomous variables were examined by frequency distribution and recorded as proportions for both groups, RTE and non-RTE. The Mann–Whitney *U* test was used to compare age at diagnosis between groups. Univariate analyses using Kaplan-Meier survival were used to estimate the fraction of patients free of RTE over time after the diagnosis, and inference on event-free survival distribution differences was assessed using the log-rank test. Owing to the exploratory nature of this study, differences were considered statistically significant for a *p* value < 0.05 without correction for multiple comparisons. Analyses were performed using SPSS, version 23.

## 3. Results

We identified 10 out of 54 patients with RTE and present results accordingly, as percentages (Table 1) and proportions (Table 2) with respect to the presence or absence of classical risk factors, risk profile based on serological features, and the presence of lupus anticoagulant as well as reversion to negative status.

**Figure 1:**
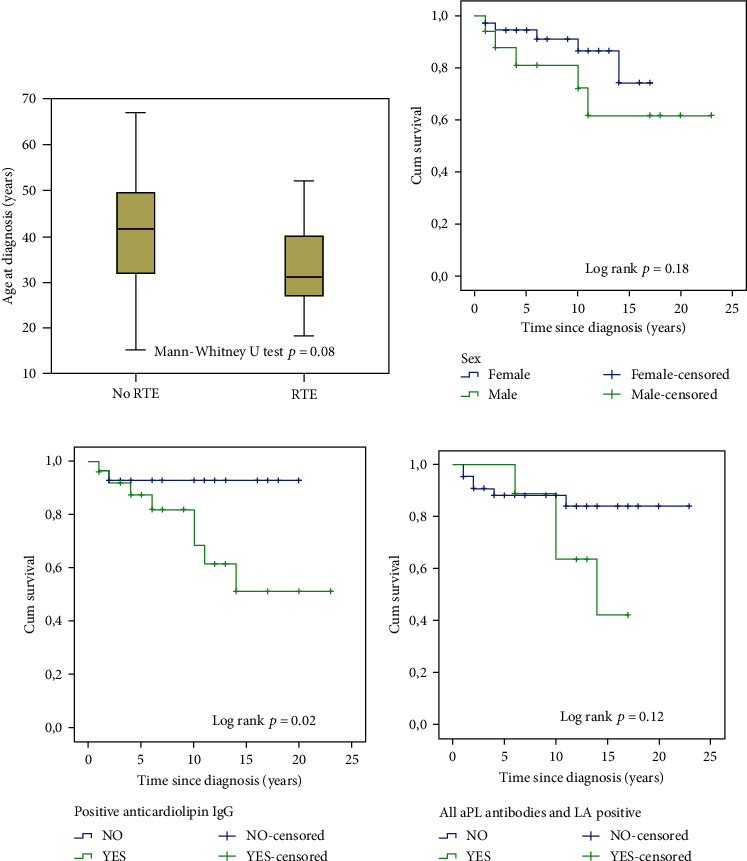
Shown are differences between repeated thrombotic event (RTE) and non-RTE groups regarding: age at diagnosis (a), Kaplan-Meier curves where the event is “the occurrence of a RTE after an initial PAPS diagnosis” for sex (b), positive anti-cardiolipin IgG antibody (c), and full aPL and LA positivity (d).

Overall, one-third of the patients were male and 93% of the overall group was Caucasian, with a median age of 40 years at diagnosis, followed for a median time of 11.5 years. The median age for the group who had RTE at the time of diagnosis was ten years younger than the remainder (non-RTE) (Figure 1(a)), with a higher proportion of males in the RTE group (Figure 1(b)). Rethrombosis occurred a median of five years from diagnosis.

At onset of APS diagnosis, the vast majority of thrombotic events were considered unprovoked, and the type, seriousness, and recurrence of thrombotic events, incidence of hypertension, dyslipidaemia, type 2 diabetes mellitus, obesity and the proportion with aGAPSS ≥8 were slightly different in the two groups. None of the RTE patients had been on hormonal contraception, immobile, or in a perioperative condition. All females stopped hormonal contraception upon PAPS diagnosis and remained free of further clotting events. There was not enough data to evaluate INR immediately prior or at the time of the thrombotic event in the RTE group. More specifically, the TTR in the past year was unknown for one-third of our patients and found to be > than 75% in only 2/6 RTE (33%) and 23/40 non-RTE (57.5%).

Positivity for ACA IgG, LA, and all aPL subtypes was more frequent in the RTE group, highlighted when time in follow-up was considered (Figures 1(c) and 1(d), respectively). At the end of follow-up the RTE group mostly remained LA (80%), anticardiolipin IgG and anti-*β*(2)GPI IgG (70%) positive with a higher proportion of RTE patients also fitting to a high-risk profile category (80%). In addition, reversion to negative status was infrequent, observed in 20% of RTE vs. 25% of non-RTE patients. Only one non-RTE patient anticoagulated for 11 years stopped warfarin, with no RTE over three years of follow-up. This patient had reverted to negative serological status, had stopped smoking at the time of diagnosis, and had no other classical risk factors for thrombosis. Hydroxychloroquine treatment was followed by 11/44 (25%) non-RTE and 3/10 (33%) RTE patients; 3/44 (7%) non-RTE but no RTE patients were also on low-dose aspirin.

## 4. Discussion

Modern concepts of clinical judgement were applied in the diagnosis of PAPS in our patient cohort with a median age of 40 years and a high frequency of an aGAPSS score ≥8 [15]. Judging from the number, recurrence and characteristics of the thrombotic events, our patients fulfilled robust characteristics for PAPS. The overall thrombosis recurrence rate of 18.5% (10/54 patients) at 11.5 years is not unexpected when considering that a longer follow-up study reports a rethrombosis rate of 44% at 18 years [19].

In comparison to non-RTE, the RTE group was characterized by younger patients, male sex, and positivity for all laboratory markers more specifically for ACA IgG at onset, as well as a sustained high-risk profile based on APS laboratory markers. Our results are supported by a previous study that found male sex and elevated baseline ACA IgG to predict rethrombosis [20]. The small number of patients in our cohort did not allow for statistical considerations as regards onset comorbidities and recurrent thrombosis.

In both groups, a minority of patients reverted to negative status along follow-up. Our results are in line with the previous observation that aPL profiles are stable in 80% of the patients, more frequently observed in LA or triple aPL positivity over a median follow-up of 5 years [9].

The fact that INR control over time was unknown for most of our patients including those with RTE, some of whom may have resorted to advice outside the Anticoagulation Clinic, does not allow us to draw conclusions over the effectiveness of anticoagulation in either group of patients, which we recognize as difficult to achieve even when fully compliant. The main questions thus arising from our study are as follows: is there a possibility that some of the APS patients who never suffered RTE along follow-up and reverted to a negative status have inactive disease? Could this group of patients safely stop anticoagulation? To date, available evidence is still limited [21, 22].

## 5. Conclusion

In our study, at initial presentation, PAPS patients with RTE have unique characteristics, which nevertheless require confirmation, due to our study limitations. Current recommendations for a PAPS diagnosis do not account for information about the individual's natural history of the disease. Introducing a concept of disease activity might allow for optimization of treatment choices along follow-up. Prospective regular clinical and laboratory monitoring of patients with PAPS may be warranted, hopefully contributing towards more confident diagnostic, monitoring, and therapeutic guidelines, thus sparing patients from unnecessary lifelong anticoagulation.

## Figures and Tables

**Table 1:** Demographic, clinical, and laboratory parameters and risk profile at onset and end of follow-up, represented as frequencies, according to the occurrence of repeated thrombotic events in patients under anticoagulation.

Parameters	Overall (*n* = 54), *n* (%)	(a) Recurrent thrombotic events (RTE) under anticoagulation (*n* = (10))	(b) No thrombotic events under anticoagulation (*n* = (44))
*At onset:*			
Age (*y*), median (IQR) at diagnosis	40 (31–49)	31 (26–41)	41.5 (32–50)
Male, *n* (%)	17 (31)	5 (50)	12 (27)
Caucasian, *n* (%)	50 (93)	10 (100)	40 (91)
Arterial hypertension, *n* (%)	23 (43)	3 (30)	20 (45.5)
Dyslipidaemia, *n* (%)	21 (39)	6 (60)	15 (34)
Active smoking	21 (39)	4 (40)	17 (39)
Type 2 diabetes mellitus, *n* (%)	6 (11)	1 (10)	5 (11)
Obesity, *n* (%)	14 (36)	4 (40)	10 (23)
Hormonal contraception, *n* (%)	10 (28)	0	10 (23)
Immobility	5 (9)	0	5 (11)
Perioperative	1 (2)	0	1 (2)
Single ab positive, *n* (%)	23 (43)	2 (20)	21 (48)
Double ab positive, *n* (%)	9 (17)	1 (10)	8 (18)
Triple positive, *n* (%)	22 (41)	7 (70)	15 (34)
LA, *n* (%)	50 (93)	10 (100)	40 (91)
Anticardiolipin IgG, *n* (%)	26 (48)	8 (80)	18 (41)
Anti-*β*(2)GPI IgG, *n* (%)	26 (48)	7 (70)	19 (43)
High-risk profile at onset, *n* (%)	49 (91)	10 (100)	39 (88)
aGAPSS ≥8, *n* (%)	40 (74)	8 (80)	32 (73)
Clotting events prior to APS diagnosis:			
Recurrent, *n* (%)	22 (41)	3 (30)	19 (43)
Potentially life-threatening, *n* (%)	30 (56)	7 (70)	23 (52)
Severe arterial, *n* (%)	13 (24)	2 (20)	11 (25)
Severe venous, *n* (%)	18 (33.3)	5 (50)	13 (29.5)
Time after diagnosis when clotting event reoccurred after APS diagnosis (*y*), median (IQR)	—	5 (1.75–10.25)	NA
Type of event:			
DVT, *n*		4	NA
PE, *n*		3	NA
Arterial, *n*		3	NA
INR within therapeutic range when clotting event recurred after APS diagnosis, *n* (%)	—	2/2 (INR unknown for 8 patients)	NA
% time in therapeutic range >75%, in the last year of follow-up *n* (%)	25/46 (54) ^∗^ data not known in 18 patients	2/6 (33) ^∗^ data not known in 4 patients	23/40 (57.5) ^∗^ data not known in 14 patients
*At end of follow-up:*			
Follow-up (*y*), median (IQR)	11.5 (6–16)	13 (9–19.5)	11 (5–14)
Number of aPL and LA measurements over last three years of follow-up, median (IQR)	3 (2–3)	2.5 (2–3)	3 (2–3)
LA, *n* (%)	30 (56)	8 (80)	22 (50)
Anticardiolipin IgG, *n* (%)	22 (41)	7 (70)	15 (34)
Anti-*β*(2)GPI IgG, *n* (%)	20 (37)	7 (70)	13 (29.5)
Sustained high-risk profile, *n* (%)	31 (57)	8 (80)	23 (59) ^∗^ data not known in 5 patients
Reversion to negative status (aPL and LA), *n* (%)	13 (24)	2 (20)	11 (25)
Time after APS diagnosis (years) at which reversion to negative status (aPL and LA) was detected, median (IQR)	3 (0–9)	3 (0–9)	7 (0–8)

**Table 2:** Demographic, clinical, and laboratory parameters and risk profile at onset and end of follow-up, according to the occurrence or not of repeated thrombotic events in patients under anticoagulation, presence of the characteristic versus absence of the characteristic (%).

Characteristic	Total patients with the characteristic, *n* (%)	Patients with repeated thrombotic events under anticoagulation (*n* = 10), presence of the characteristic vs. absence of the characteristic (%) ^∗^
Male	17 (31)	29 vs. 13.5
Caucasian	50 (93)	20 vs. 0
Risk factors at the time of APS diagnosis:		
Hypertension	23 (43)	13 vs. 22.5
Dyslipidaemia	21 (39)	28.5 vs. 12
Active smoking	21 (39)	19 vs. 18
Type 2 diabetes mellitus	6 (11)	17 vs. 19
Hormonal contraception	10 (18.5)	0 vs. 23
Obesity	14 (26)	29 vs. 15
Immobility	5 (9)	0 vs. 10
Perioperative	1 (2)	0 vs. 19
aGAPSS ≥8, *n* (%)	40 (74)	20 vs. 14
*At onset:*		
Anticardiolipin IgM positive	14 (26)	29 vs. 15
Anticardiolipin IgG positive	26 (48)	31 vs. 7
Anti-*β*(2)GPI IgM positive	15 (28)	33 vs. 13
Anti-*β*(2)GPI IgG positive	26 (48)	27 vs. 11
Lupus anticoagulant positive	50 (93)	20 vs. 0
Lupus anticoagulant alone positive	18 (33)	11 vs. 18
LA and all other aPL	9 (17)	44 vs. 13
High-risk profile at onset, *n* (%)	49 (91)	20 vs. 0
*At end of follow-up:*		
Anticardiolipin IgM positive	7 (13)	28 vs. 17
Anticardiolipin IgG positive	22 (41)	32 vs. 9
Anti-*β*(2)GPI IgM positive	4 (7)	75 vs. 14
Anti-*β*(2)GPI IgG positive	20 (37)	35 vs. 9
Lupus anticoagulant positive	30 (55)	27 vs. 8
Lupus anticoagulant alone positive	13 (24)	8 vs. 22
LA and all other aPL	3 (5.5)	67 vs. 2
High-risk profile, *n* (%)	31 (57)	26 vs. 9
Sustained high-risk profile status, *n* (%)	31 (57)	26 vs. 9
Nonreversion to negative status (aPL and LA), *n* (%)	41 (76)	19.5 vs. 16

^∗^Ratio of RTE patients positive for a characteristic over the number of patients in the cohort with that characteristic versus (vs.) ratio of RTE patients without that characteristic over the number of characteristic negative patients in the cohort.

## Data Availability

Readers can access the data supporting the conclusions of the study through a supplementary document entitled Working Database.

## Additional Points

*Key Message*. Assertive lifelong treatment of patients with primary antiphospholipid antibody syndrome might warrant prospective regular clinical and laboratory monitoring.
